# Evaluation of Plasma Extracellular Vesicle MicroRNA Signatures for Lung Adenocarcinoma and Granuloma With Monte-Carlo Feature Selection Method

**DOI:** 10.3389/fgene.2019.00367

**Published:** 2019-04-26

**Authors:** Xiangbo Chen, Yunjie Jin, Yu Feng

**Affiliations:** ^1^Key Laboratory of Molecular Epigenetics of the Ministry of Education, Northeast Normal University, Changchun, China; ^2^Hangzhou Baocheng Biotechnology Co., Ltd., Hangzhou, China; ^3^Department of Oncology, Shanghai Putuo People’s Hospital, Shanghai, China; ^4^Shuguang Hospital, Shanghai University of Traditional Chinese Medicine, Shanghai, China

**Keywords:** microRNA signatures, biomarker, classification, lung adenocarcinoma, granuloma

## Abstract

Extracellular Vesicle (EV) is a compilation of secreted vesicles, including micro vesicles, large oncosomes, and exosomes. It can be used in non-invasive diagnosis. MicroRNAs (miRNAs) processed by exosomes can be detected by liquid biopsy. To objectively evaluate the discriminative ability of miRNAs from whole plasma, EV and EV-free plasma, we analyzed the miRNA expression profiles in whole plasma, EV and EV-free plasma of 10 lung adenocarcinoma and 9 granuloma patients. With Monte-Carlo feature selection method, the top discriminative miRNAs in whole plasma, EV and EV-free plasma were identified, and they were quite different. Using the Repeated Incremental Pruning to Produce Error Reduction (RIPPER) method, we learned the classification rules: in whole plasma, granuloma patients did not express hsa-miR-223-3p while the lung adenocarcinoma patients expressed hsa-miR-223-3p; in EV, the hsa-miR-23b-3p was highly expressed in granuloma patients but not lung adenocarcinoma patients; in EV-free plasma, hsa-miR-376a-3p was expressed in granuloma patients but barely expressed in lung adenocarcinoma patients. For prediction performance, whole plasma had the highest weighted accuracy and EV outperformed EV-free plasma. Our results suggested that EV can be used as lung cancer biomarker. However, since it is less stable and not easy to detect, there are still technological difficulties to overcome.

## Introduction

Blood is a mixture of plasma, blood platelet and various blood cells, such as erythrocytes, leukocytes, neutrophilic granulocytes, eosinophilic granulocytes, basophilic granulocytes, monocytes, and lymphocytes (Basu and Kulkarni, 2014). It can reflect the body health and wellness. Extracellular Vesicle (EV) is a compilation of secreted vesicles, including micro vesicles, large oncosomes, and exosomes (Lawson et al., 2018). Exosomes, with a diameter of 30–100 nm, are a kind of membrane-bound EVs and originate from endosome (Raposo and Stoorvogel, 2013). Nearly all kinds of cells can secrete exosomes whether under normal or stressful conditions (Srivastava et al., 2015). When compared with normal cells, tumor cells of the specific organs have been proven to secrete more exosomes. Besides, the membrane of exosomes richly contains plenty of functional proteins, including tetraspanin, endosome-related membrane transport and fusion proteins and multivesicular bodies-genesis proteins, and thus exosomes could be applied as biomarkers (O’Driscoll, 2015). Exosomes can be extracted from diverse body fluids, which contain numerous biological molecules (DNAs, RNAs, and proteins). Recently, liquid biopsy has been developed as a novel, non-invasive diagnosis method to explore tumor development (Sheridan, 2016).

MicroRNAs (miRNAs) processed by exosomes could be detected by liquid biopsy (Iranifar et al., 2019). miRNAs are a group of non-coding RNAs, which regulate gene expression at the post-transcriptional and translational levels (Inamura, 2017). Dysregulation of miRNA expression is related to the progression of lung adenocarcinoma. Besides, Nadal et al. (2014) have demonstrated that different morphological subtypes of lung adenocarcinoma have specific miRNA expression profiles, for instance, miR-212-3p, miR-132-5p, and miR-27a-3p are found significantly upregulated in adenocarcinomas with solid subtype. A mass of miRNAs play important roles in the process of lung cancer pathogenesis and are recognized as potential diagnostic biomarkers and tumor targeted therapeutic molecules (Inamura, 2017).

As a well-studied, common cancer, lung cancer maintains the leading cause of cancer-specific death around the world. Adenocarcinoma accounts for nearly half of all lung cancer types, remaining the most common histologic subtype (Travis et al., 2011; Rosell and Karachaliou, 2018). Although the development of new therapies has significantly improved the prognosis of patients with lung adenocarcinoma, the 5-year survival rate remains low (less than 16%) (Crino et al., 2010).

To evaluate the discriminative ability of miRNAs from whole plasma, EV, and EV-free plasma, we analyzed the miRNA expression profiles in whole plasma, EV, and EV-free plasma of lung adenocarcinoma and granuloma patients. The same feature selection method, Monte-Carlo feature selection and the same rule learner, Repeated Incremental Pruning to Produce Error Reduction (RIPPER), were applied in the three miRNA expression datasets for lung adenocarcinoma and granuloma patients. The prediction performances and classification rules of whole plasma, EV, and EV-free plasma were compared and analyzed. Our results suggested that the prediction performance of EV miRNAs was better than EV-free plasma miRNAs. What’s more, we identified EV specific miRNA expression pattern in lung cancer. These results supported the usage of EV miRNAs as lung cancer biomarkers but the whole plasma achieved a better prediction performance. The utilization of EV biomarkers still has a long way to go.

## Materials and Methods

### The MicroRNA Expression Profiles in Whole Plasma, EV, and EV-Free Plasma

We downloaded the processed miRNA expression profiles in whole plasma, EV, and EV-free plasma of 10 lung adenocarcinoma patients and the miRNA expression profiles in whole plasma, EV and EV-free plasma of 9 granuloma patients from GEO (Gene Expression Omnibus) under accession number of GSE71661 on August 30, 2018. The expression levels of miRNAs were measured with next generation sequencing using Illumina HiSeq 2500. The reads were mapped onto known human miRNA in miRbase Release 21 using Blast and Bowtie. The mapped reads were normalized with the total number of reads. In each miRNA dataset of whole plasma, EV, and EV-free plasma, there were 10 lung adenocarcinoma and 9 granuloma patients; there were 1,509 miRNAs. The downloaded miRNA profiles were provided in Supplementary Table S1.

To systematically compare the miRNA expression difference between lung adenocarcinoma and granuloma patients, whole plasma, EV, and EV-free plasma were analyzed separately. Our goal was to compare their prediction performance and unique expression of miRNAs.

### Key MicroRNAs in Whole Plasma, EV, and EV-Free Plasma Identified With Monte-Carlo Feature Selection

Since there were 19 samples and 1,509 miRNA features in whole plasma, EV, and EV-free plasma dataset, the number of features was much greater than the sample size. If we use all miRNAs to build the classification model, all samples will be perfectly classified. But it will be overfitting and will have no actual meanings. Therefore, we adopted the Monte-Carlo feature selection (Draminski et al., 2008) to identify the key miRNA features and then used these few key features to construct the classification model. The Monte-Carlo feature selection method has been widely used and has achieved great performance in many fields (Chen et al., 2018c,e; Pan et al., 2019).

The Monte-Carlo feature selection method will randomly choose several features multiple times and then construct a series of tree classifiers (Chen et al., 2018a; Pan et al., 2018b; Wang et al., 2018). Based on the frequency and classification accuracies of the feature nodes on these classification trees, each feature will be assigned with a relative importance. Intuitively speaking, if a feature has been selected many times to construct the classification tree, it is important as the classification tree will find the most discriminative features to be the nodes.

Let’s denote the total number of miRNA features with *d*, i.e., 1,509 in this study. *m* miRNA features (*m*≪*d*) will be randomly selected and be used to construct *t* classification trees for *s* times. Each of the *t* trees was trained and tested based on the training and test patient samples randomly divided from the full dataset. Therefore, *s* ⋅ *t* classification trees will be constructed. Based on how many times a miRNA feature *g* has been selected by these *s* ⋅ *t* trees and how much this miRNA feature *g* has contributed to the classification of the *s* ⋅ *t* trees, its relative importance (RI) can be calculated:

(1) $${\text{RI}}_{\text{g}}=\sum_{\tau =1}^{\text{st}}{\left(\mathrm{wAcc}\right)}^{\text{u}}\sum_{{\text{n}}_{\text{g}}(\tau )}\text{IG}\left(n_{\text{g}}(\tau )\right){\left(\frac{\text{no}\cdot \text{in }n_{\text{g}}(\tau )}{\text{no}\cdot \text{in}\tau }\right)}^{\text{v}}$$

where *wAcc* is the weighted classification accuracy of decision tree *τ*, IG(*n*_g_(*τ*)) is the information gain of node *n*_g_(*τ*), which is a decision rule using the expression levels of miRNA feature *g*, (no ⋅ in *n*_g_(*τ*)) is the number of samples under node *n*_g_(*τ*), (no ⋅ in *τ*) is the number of samples in decision tree *τ*, *u*, and *v* are adjust parameters.

By analyzing the *s* ⋅ *t* classification trees, each miRNA feature will be assigned with a RI and will be ranked decreasingly.

The Monte-Carlo feature selection method was applied using the dmLab software (Draminski et al., 2008) downloaded from http://www.ipipan.eu/staff/m.draminski/mcfs.html.

### Classification Rules for Lung Adenocarcinoma and Granuloma in Whole Plasma, EV, and EV-Free Plasma Learned With RIPPER

Repeated Incremental Pruning to Produce Error Reduction is a widely used method to learn the classification rules (Cai et al., 2018; Chen et al., 2018a,c,e,f; Pan et al., 2018a). Since we want to evaluate the prediction performance objectively, we did the 10-fold cross-validation for three times and combined the three-time results. In each cross validation (Wang et al., 2017; Zhang et al., 2017; Chen et al., 2018b,d; Li et al., 2018), the samples were randomly divided into 10 parts and each part was used as test dataset for once. After 10 rounds, all samples have been tested. As the random splits of data may cause bias, we repeated the 10-fold cross-validation for three times. In this study, the lung adenocarcinoma patients and granuloma patients were treated as positive samples and negative samples, respectively. We used weighted accuracy to evaluate the RIPPER prediction performance, i.e., the average of the accuracies of positive samples and negative samples.

## Results

### The Discriminative MicroRNAs Between Lung Adenocarcinoma and Granuloma Patients in Whole Plasma, EV, and EV-Free Plasma

**FIGURE 1:**
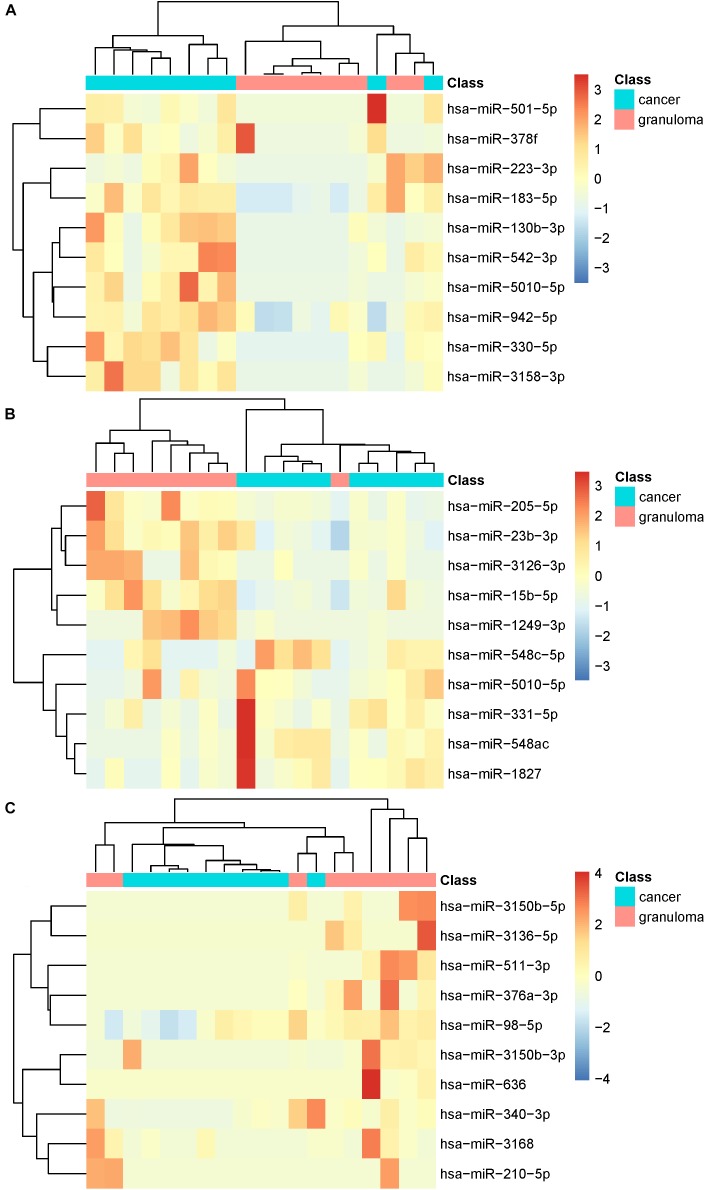
The heatmaps of top 10 microRNAs in whole plasma, EV, and EV-free plasma. **(A)** For whole plasma, there were two miss clustered cancer patients; **(B)** for EV, there was one miss clustered granuloma patient; **(C)** for EV-free plasma, the cluster pattern was not clear.

The miRNA expression profiles of lung adenocarcinoma and granuloma patients in whole plasma, EV and EV-free plasma were analyzed separately. In whole plasma, the top 10 discriminative miRNAs were hsa-miR-223-3p, hsa-miR-501-5p, hsa-miR-130b-3p, hsa-miR-5010-5p, hsa-miR-330-5p, hsa-miR-378f, hsa-miR-3158-3p, hsa-miR-542-3p, hsa-miR-183-5p and hsa-miR-942-5p. In EV, the top 10 discriminative miRNAs were hsa-miR-23b-3p, hsa-miR-548ac, hsa-miR-3126-3p, hsa-miR-15b-5p, hsa-miR-205-5p, hsa-miR-5010-5p, hsa-miR-331-5p, hsa-miR-1249-3p, hsa-miR-548c-5p, and hsa-miR-1827. In EV-free plasma, the top 10 discriminative miRNAs were hsa-miR-511-3p, hsa-miR-376a-3p, hsa-miR-3150b-3p, hsa-miR-3150b-5p, hsa-miR-3168, hsa-miR-98-5p, hsa-miR-3136-5p, hsa-miR-210-5p, hsa-miR-340-3p, and hsa-miR-636. Figure 1 shows the heatmaps of the top 10 miRNAs in whole plasma, EV and EV-free plasma. The miRNAs and patients were clustered using ward D2 method (Murtagh and Legendre, 2014) based on Euclidean distance. The R package pheatmap^1^ was applied to plot the heatmaps. It can be seen from Figure 1 that for whole plasma, there were two miss clustered cancer patients; for EV, there was one miss clustered granuloma patient; for EV-free plasma, the cluster pattern was not clear. The miRNAs in EV-free plasma were not suitable as cancer biomarkers.

**FIGURE 2:**
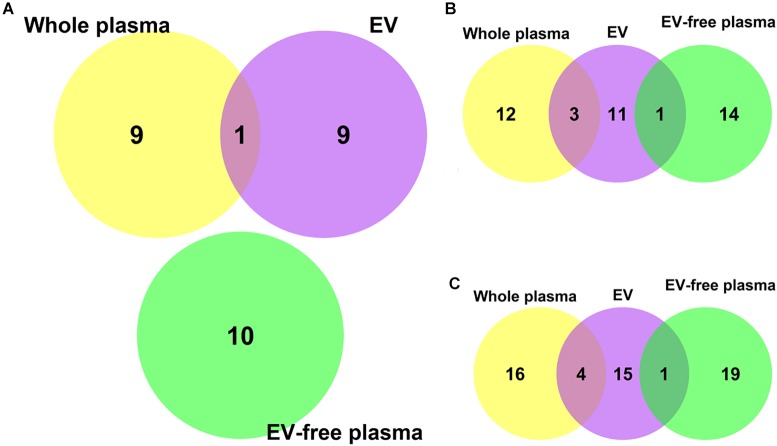
The Venn Diagram of the top 10, 15, and 20 discriminative microRNAs in whole plasma, EV and EV-free plasma. **(A)** The overlap among the top 10 microRNAs. There was only one overlapped microRNA between whole plasma and EV. The overlap microRNA was hsa-miR-5010-5p. **(B)** The overlap among the top 15 microRNAs. **(C)** The overlap among the top 20 microRNAs.

We plotted the Venn Diagram of the top 10 discriminative miRNAs in whole plasma, EV and EV-free plasma in Figure 2A. There was only one overlapped miRNA between whole plasma and EV. The overlap miRNA was hsa-miR-5010-5p. It can be seen that the miRNA expression pattern was different in whole plasma, EV and EV-free plasma. It was necessary to investigate which blood compartments should be used for biomarker discovery.

To investigate whether the overlap pattern would change when more miRNAs were analyzed, we plotted Venn Diagrams of the top 15 and top 20 miRNAs as Figure 2B,C, respectively. There was still no overlap among the whole plasma, EV and EV-free plasma. The overlap between whole plasma and EV became larger when more top miRNAs were included but the overlap between EV and EV-free plasma remained to be one no matter whether the top 15 or 20 miRNAs were analyzed. The EV miRNAs were more similar with the whole plasma miRNAs than the EV-free plasma miRNAs.

### The Prediction Accuracies of MicroRNA Signatures for Lung Adenocarcinoma and Granuloma Patients in Whole Plasma, EV, and EV-Free Plasma

We evaluated the prediction accuracies of miRNA signatures for lung adenocarcinoma and granuloma patients in whole plasma, EV and EV-free plasma with 10-fold cross validations. To avoid the bias of random splits of samples, we repeated the 10-fold cross validation for three times. Therefore, the samples size in the confusion matrix will be the original sample size 19 multiplied by 3 which was 57.

The confusion matrices of miRNA signatures in whole plasma, EV and EV-free plasma were given in Table 1. The weighted accuracies using whole plasma, EV and EV-free plasma miRNA data were 77.22, 65.19, and 64.82%, respectively. The EV miRNAs performed better than the EV-free plasma miRNAs. The accuracy of granuloma in EV-free plasma, 29.63%, was extremely low.

**Table 1:** The confusion matrices of whole plasma, EV, and EV-free plasma microRNAs.

Whole plasma			EV			EV-free plasma		
	Predicted granuloma	Predicted adenocarcinoma		Predicted granuloma	Predicted adenocarcinoma		Predicted granuloma	Predicted adenocarcinoma
Actual granuloma	21	6	Actual granuloma	19	8	Actual granuloma	8	19
Actual adenocarcinoma	7	23	Actual adenocarcinoma	12	18	Actual adenocarcinoma	0	30
Granuloma accuracy	21/(21+6) = 77.77%		Granuloma accuracy	19/(19+8) = 70.37%		Granuloma accuracy	8/(8+19) = 29.63%	
Adenocarcinoma accuracy	23/(7+23) = 76.67%		Adenocarcinoma accuracy	18/(12+18) = 60.00%		Adenocarcinoma accuracy	30/(0+30) = 100.00%	
Weighted accuracy	(77.77%+76.67%)/2 = 77.22%		Weighted accuracy	(70.37%+60.00%)/2 = 65.19%		Weighted accuracy	(29.63%+100.00%)/2 = 64.82%	

### The Classification Rules in Whole Plasma, EV, and EV-Free Plasma

With the RIPPER method, we learned the classifications of miRNA expression levels in whole plasma, EV and EV-free plasma. These rules were given in Table 2. In whole plasma, granuloma patients did not express hsa-miR-223-3p while the lung adenocarcinoma patients expressed hsa-miR-223-3p. In EV, the hsa-miR-23b-3p was highly expressed in granuloma patients but not lung adenocarcinoma patients. In EV-free plasma, hsa-miR-376a-3p was expressed in granuloma patients but barely expressed in lung adenocarcinoma patients. We compared the mean expression levels of hsa-miR-23b-3p in whole plasma cancer, whole plasma granuloma, EV cancer and EV granuloma. We found that in EV, hsa-miR-23b-3p was more highly expressed in granuloma than cancer with a fold change of 1.82, while in whole plasma, hsa-miR-23b-3p was more lowly expressed in granuloma than cancer with fold change of 0.84. What’s more, we compared the mean expression levels of hsa-miR-376a-3p in EV-free plasma as well. We found that in EV-free plasma, the mean expression levels of hsa-miR-376a-3p in cancer and granuloma were 0 and 10.30, respectively, while in whole plasma, the mean expression levels of hsa-miR-376a-3p in cancer and granuloma were 1.45 and 0, respectively. The expression pattern between EV or EV-free plasma and whole plasma were different. These results suggested it was necessary to measure the EV, EV-free plasma and whole plasma, separately.

**Table 2:** The RIPPER rules in whole plasma, EV and EV-free plasma.

Whole plasma		EV		EV-free plasma	
Granuloma	hsa-miR-223-3p <= 0	Granuloma	hsa-miR-23b-3p >= 210.43	Granuloma	hsa-miR-376a-3p >= 4.50
Adenocarcinoma	Others	Adenocarcinoma	Others	Adenocarcinoma	Others

hsa-miR-223-3p was reported to have an increased expression in H. pylori-infected gastric cancer patients, which was related to progressive proliferation and migration of cancer cells (Ma et al., 2014; Wang et al., 2015). Thus, in plasma, the expression of hsa-miR-223-3p in granuloma patients would not be as high as in cancer patients.

Zhou et al. (2015) found that cancer patients with higher expression of has-miR-23b had better outcomes then those with lower expression. In our study, we found that has-miR-23b-3p had higher expression in granuloma patients compared to in lung adenocarcinoma patients.

Joerger et al. (2014) reported that hsa-miR-376a was insensitive to perturbations in advanced non-small cell lung cancer patients. We found has-miR-376a-3p had a higher expression in granuloma patients, while its expression was very low in lung adenocarcinoma patients.

## Discussion

We identified the discriminative miRNAs in different blood compartments, such as hsa-miR-501-5p and hsa-miR-130b-3p in plasma; hsa-miR-548ac in EV and hsa-miR-511-3p in EV-free plasma.

hsa-miR-501 has been proven to have an association with clear cell renal cell carcinoma (Liu et al., 2018), pancreatic ductal adenocarcinoma (Liao et al., 2018), cervical cancer (Guo et al., 2018) and so on. Besides, they all found upregulation of has-miR-501 enhances tumor cell proliferation, migration and invasion.

hsa-miR-130b-3p is a novel miRNA in lung cancer, we found hsa-miR-130b-3p are upregulated in the plasma of lung cancer patients, which would be applied as a new biomarker to distinguish cancer and granuloma, and further guide therapeutic decisions clinically.

As for hsa-miR-548, Liu et al. (2015) investigated hsa-miR-548 expression in fresh tumor tissues from 22 patients with primary non-small cell lung cancer via RT-PCR and they found that the hsa-miR-548 expression level was significantly higher (*p* < 0.01) in adjacent non-tumor tissues than that in the tumor. That is, non-small cell lung cancer would down-regulate the expression of hsa-miR-548. Furthermore, they also observed that hsa-miR-548 was involved in the migration and invasion of non-small cell lung cancer cells by targeting the AKT1 signaling pathway.

For hsa-miR-511-3p, it has been reported to be related to lung adenocarcinoma by triggering BAX (Zhang et al., 2014) and TRIB2 (Zhang et al., 2012).

As for the diagnostic value, plasma is the most valuable, followed by EV and EV-free plasma. Previous studies have demonstrated that exosomes can be used as a type of novel biomarker for tumors and some benign diseases (Principe et al., 2013; Vella et al., 2016). Considering the diagnostic value of testing plasma is better than testing exosomes in plasma, many useful information may be missed when only exosomes in plasma were tested. The reasons are as follows: (1) Methods like OptiPrep^TM^ density-based separation (DG-Exos), ultracentrifugation (UC-Exos), and immunoaffinity capture using anti-EpCAM-coated magnetic beads (IAC-Exos) are not effective enough to isolate exosomes and may destroy exosomes during the isolation process (Greening et al., 2015); (2) exosomes are not stable and are easily degraded, which could cause a bias (Kumar et al., 2018).

Since the sample size of this study was limited, the results should be validated in an independent large cohort. Another factor that may have affected the results was the disease type. For lung adenocarcinoma, the results were like this. But for other diseases, which release a large amount of RNAs and proteins into the circulatory system directly, the importance of exosome may decrease.

## Conclusion

Extracellular Vesicle is a promising technology for non-invasive diagnosis. miRNAs processed by exosomes can be detected by liquid biopsy and used as biomarkers. To evaluate the discriminative ability of miRNAs from whole plasma, EV and EV-free plasma, we analyzed the miRNA expression profiles in whole plasma, EV and EV-free plasma of lung adenocarcinoma and granuloma patients. We found that the top discriminative miRNAs in whole plasma, EV and EV-free plasma were quite different, and the classification rules also varied. The prediction performance of whole plasma was the best but the EV outperformed EV-free plasma. Our results suggested that EV can be used as a lung cancer biomarker but EV may be less stable or difficult to detect than whole plasma, therefore, the whole plasma was still a good choice as lung cancer signatures.

## Author Contributions

XC and YJ did the conception and design, performed the sample collection, analyzed and interpreted the data, and wrote, reviewed, and/or revised the manuscript. XC oversaw the developmental methodology. All authors read and approved the final manuscript.

## Conflict of Interest Statement

XC was employed by company Rongze Biotechnology Co., Ltd. The remaining authors declare that the research was conducted in the absence of any commercial or financial relationships that could be construed as a potential conflict of interest.

## Footnotes

1 https://CRAN.R-project.org/package=pheatmap

## Supplementary Material

TABLE S1 The processed microRNA expression profiles.
